# The critical role of toll-like receptor 4 in bone remodeling of osteoporosis: from inflammation recognition to immunity

**DOI:** 10.3389/fimmu.2024.1333086

**Published:** 2024-03-05

**Authors:** Xianping Zhu, Li Du, Lai Zhang, Lingzhi Ding, Weifang Xu, Xuezheng Lin

**Affiliations:** ^1^ Department of Orthopedics, Taizhou Central Hospital (Taizhou University Hospital), Taizhou, Zhejiang, China; ^2^ Educational Administration Department, Chongqing University Cancer Hospital, Chongqing, China; ^3^ Department of Orthopedics, Taizhou Municipal Hospital, Taizhou, Zhejiang, China; ^4^ Department of Anesthesia Surgery, Taizhou Central Hospital (Taizhou University Hospital), Taizhou, China

**Keywords:** osteoporosis, TLR4, osteoblast, osteoclasts, treatment, mechanism

## Abstract

Osteoporosis is a common chronic metabolic bone disorder. Recently, increasing numbers of studies have demonstrated that Toll-like receptor 4 (TLR4, a receptor located on the surface of osteoclasts and osteoblasts) plays a pivotal role in the development of osteoporosis. Herein, we performed a comprehensive review to summarize the findings from the relevant studies within this topic. Clinical data showed that TLR4 polymorphisms and aberrant TLR4 expression have been associated with the clinical significance of osteoporosis. Mechanistically, dysregulation of osteoblasts and osteoclasts induced by abnormal expression of TLR4 is the main molecular mechanism underlying the pathological processes of osteoporosis, which may be associated with the interactions between TLR4 and NF-κB pathway, proinflammatory effects, ncRNAs, and RUNX2. *In vivo* and *in vitro* studies demonstrate that many promising substances or agents (i.e., methionine, dioscin, miR-1906 mimic, artesunate, AEG-1 deletion, patchouli alcohol, and Bacteroides vulgatus) have been able to improve bone metabolism (i.e., inhibits bone resorption and promotes bone formation), which may partially attribute to the inhibition of TLR4 expression. The present review highlights the important role of TLR4 in the clinical significance and the pathogenesis of osteoporosis from the aspects of inflammation and immunity. Future therapeutic strategies targeting TLR4 may provide a new insight for osteoporosis treatment.

## Introduction

Osteoporosis, a chronic metabolic bone disorder prevalent among the elderly, is characterized by a decline in bone mineral density (BMD) and compromised bone micro-architecture (1). A recent meta-analysis showed that the global prevalence of osteoporosis was 19.7% (95% CI: 18% to 21%) (2). The prevalence of osteoporosis is higher in developing countries than in developed countries (22.1% vs 14.5%) (2). The condition is closely linked to an elevated risk of bone fractures, leading to disability and mortality among affected individuals (3). With aging populations worldwide, the global economic and health burden of osteoporosis is increasing. Therefore, how to diagnose and treat osteoporosis early and accurately has become one of the major challenges in the healthcare research industry. The development of osteoporosis is a complex process involving multiple factors, such as increased bone resorption, decreased bone formation, abnormal hormone levels, malnutrition, genetic factors, chronic diseases and specific medications, low body weight, and physical inactivity (4–6). Commonly used adjunctive tests for osteoporosis include bone densitometry (dual-energy X-ray absorptiometry, quantitative computed tomography, and peripheral bone quantitative computed tomography), bone-turnover markers, and magnetic resonance imaging (7, 8). Since no definitive cure is available for treating benign osteopetrosis, fracture prevention is a hotspot of osteoporosis treatment. Calcium and vitamin D supplementation are necessary as treatments for osteoporosis, while the main drugs currently available include estrogen, selective estrogen receptor modulators (e.g., raloxifene), calcitonin, bisphosphonates (e.g., alendronate, risedronate, ibandronate, and zoledronic acid), parathyroid hormone analogue (e.g., teriparatide), and receptor activator of nuclear factor-kB ligand (RANKL) monoclonal antibody (e.g., denosumab) (9, 10). The current prevention and treatment of osteoporosis mainly emphasizes drug prevention, but drugs cannot bring the bone remodeling process back to balance. For example, bisphosphonate is used to prevent osteoporosis, but the risk of atypical femur fracture increases with the prolongation of bisphosphonate use (11). Therefore, new targets for osteoporosis prevention and treatment have become an important endeavor. In recent years, increasing numbers of studies have demonstrated that the Toll-like receptor 4 (TLR4)/nuclear factor-κB (NF-κB) pathway plays a pivotal role in the development of osteoporosis (12).

The TLR4/NF-κB pathway is a key signaling pathway in the immune system for detecting and responding to infection, inflammation, and external stimuli (13). This pathway involves multiple molecules and cytokines, including the TLR4 receptor, cascade-activated proteins in the pathway, and ultimately nuclear factor-κB (NF-κB), which is critical for the regulation of inflammation and immune responses (14). Senescence is a major risk factor for osteoporosis development (15), which can affect the gut microbial composition, cellular autophagy, and iron metabolism. These pathological alterations can induce the senescence of bone marrow mesenchymal stem cells (BMSCs) by regulating the body’s immunity, metabolic pathways, inflammation, P53 expression, and ROS production (16). TLR4 is a key member of the classical inflammatory signaling pathway. It can be activated by specific exogenous substances (i.e., bacterial endotoxin), thus prompting the activation of NF-κB into the nucleus, releasing a variety of inflammatory factors, such as TNF-α, IL-1β, and IL-6 (17). Elevation of these inflammatory mediators inhibits osteogenic differentiation of BMSCs and accelerates the maturation of osteoclasts, and ultimately leads to the occurrence of osteoporosis (12). TLR4 is expressed on the surface of osteoclasts and osteoblasts, which regulates the immune response in patients with chronic inflammation, eventually causing the onset of osteoporosis.

Since no relevant review article is available on this topic, we collected the current knowledge on the essential roles of TLR4 in osteoporosis by conducting a comprehensive review, which may be instructive to help the investigators fully understand the biological functioning of TLR4 in osteoporosis development.

## Biological profile of TLR4

TLRs (Toll-like receptors) are pattern recognition receptors (PRRs) embedded in the innate immune system that help regulate inflammation and immune response (18). TLRs belong to a series of transmembrane proteins that recognize specific molecular patterns associated with pathogens, i.e., lipoproteins, bacterial DNA, lipopolysaccharide (LPS), and double-stranded RNA (19, 20). Pattern recognition receptors recognize pathogenic microorganisms as the first step in the host’s innate immune response. TLRs play an important role in the host’s defense against infections (i.e., inflammatory response) and in triggering the immune response (i.e., recognition of pathogen-associated molecular patterns, transmembrane and intracellular TLRs, signal transduction, antigen presentation, and immunological memory) (21). These receptors are commonly expressed in several immune cell types, including macrophages, dendritic cells, neutrophils, and natural killer cells (22).Upon activation, TLRs trigger a cascade of signaling events, such as NF-B activation, which increases proinflammatory cytokines, chemokines, and other antimicrobial proteins (23).

The structure of TLR4 comprises three separate components, a leucine-rich repeat domain located on the outer membrane, an intracellularly located Toll/IL-1 receptor (TIR) domain, and a transmembrane domain (24). The three domains have different biological functions. The leucine-rich repeat domain functions for the recognition of LPS. LPS, a key component of the outer membrane of gram-negative bacteria, can lead to an inflammatory response and promote the activity of osteoclasts (25). The TIR domain is a response for transmitting extracellular signals into the cell. The transmembrane domain anchors TLR4 to enable special biological functions (26). In line with the TLR family, TLR4 is also expressed predominantly within the cellular membranes of immune cells, which participates in innate immunity *in vivo* (27). TLR4 is the main receptor known to interact with LPS. The interaction of TLR4 with gram-negative bacterial endotoxin LPS induces the activation of the TLR4 signaling cascade (28).

TLRs (i.e., TLR1, TLR2, and TLR4) can recognize and bind to multiple and diverse damage-associated molecular pattern molecules (DAMPs) and pathogen-associated molecular patterns (PAMPs), i.e., LPS, oxidized low-density lipoprotein (LDL), hyaluronic acid, heparan sulfate, free fatty acids, and heat shock protein 60 (HSP60) (29). DAMPs can be expressed by the host, probably by insertions of specific amino acids conferring ligand specificity. Endogenous DAMPS, the host-derived molecules generated by damaged tissues, can activate TLR4 receptors in osteoblasts when mechanical loading occurs. DAMPs can be expressed by the host, probably by insertions of specific amino acids conferring ligand specificity (29). In bone research, it has been reported that TLR4 can recognize DAMPS-associated musculoskeletal pathologies. DAMPs are the endogenous TLR4 ligands. They are secreted by necrotic cells and extracellular matrix (ECM) in response to tissue injury, which affects osteoclastogenesis under complex mechanisms of action. For signaling, DAMPs correlated to TLRs mainly as homodimers and heterodimers (30). Besides being able to recognize a wide range of molecules, TLR4 is capable for forming heterodimers with other TLRs, as well as interacting with several coreceptors and accessory molecules (29). There are several co-receptors that are involved in increasing responsiveness or the ability to form favorable heterodimers, such as MD-2, CD14, and CD36 (31). It was found that heterodimer TLR4-TLR6 that bound oxidized LDL, while TLR4 homodimer could not recognize oxidized LDL as an agonist.

LPS-TLR4 interaction induces a significant secretion of pro-inflammatory mediators (32). In pathogenic infections, TLR4 activation is closely associated with inflammation, autophagy, and nitrogen oxidative stress (33). In its subsequent triggering of the MyD88-dependent signaling pathway, the membrane-anchored TLR4 receptor first interacts with tTIR domain-containing adapter protein (TIRAP), a junction protein in the TLR signaling pathway that has been shown to play a role downstream of TLR4 (34). TLR4-bound TIRAP recruits MyD88 and triggers inflammatory signaling cascades, including phosphorylation of IkB and NF-kB-mediated inflammatory responses. Under normal physiological conditions, NF-kB is inhibited by IkB. Phosphorylation of IkB promotes ubiquitination and degradation of the proteasome, relieving its inhibitory effect on NF-kB, which subsequently enters the nucleus and activates the expression of a series of inflammatory mediators, thus further stimulating the inflammatory response. Aberrant regulation of the TLR4 signaling cascade is found to be correlated to many conditions, i.e., infectious diseases, metabolic disorders, immune diseases, endocrine diseases, acute organ injury, and drug addiction (35). Since osteoporosis is a metabolic and endocrine disorder, TLR4 may play an important role in the pathogenesis of this disease.

TLR4 is closely associated with osteogenic differentiation through the activation of the TLR signaling pathway. As reported, TLR4 activation on cell proliferation might be due to the variation in TLR4 agonists concentrations (36). There is evidence that a catabolic and anabolic process is associated with TLR4 activation in mesenchymal stem cells, osteoblasts, and osteoclasts (29). Through glycosylation and phosphorylation, post-translational modifications have been shown to be responsible for activation and localization of TLR4. Though remains inconclusive, TLR4 has been described to be activated in high glucose and high-fat environments, which induced osteogenic dysfunction (37). Also, TLR4 can be activated by LPS and ovariectomized surgery. Mechanistically, overexpressed Zinc finer E-box binding homeobox 1 (ZEB1), down-regulated KDM4A, overexpressed miR-137, overexpression of circ_0134944, and inhibition of miR-630 (12, 38, 39). Rajpar et al. (40) reported that mechanical loading of osteoblasts enhanced the activation of TLR4 receptors by endogenous DAMPS and/or TLR4 agonists. Taken together, TLR4 can be activated by many factors, which is correlated to osteoclastogenesis, inflammation, and immune responses.

## Literature search for identifying the eligible studies

**Figure 1:**
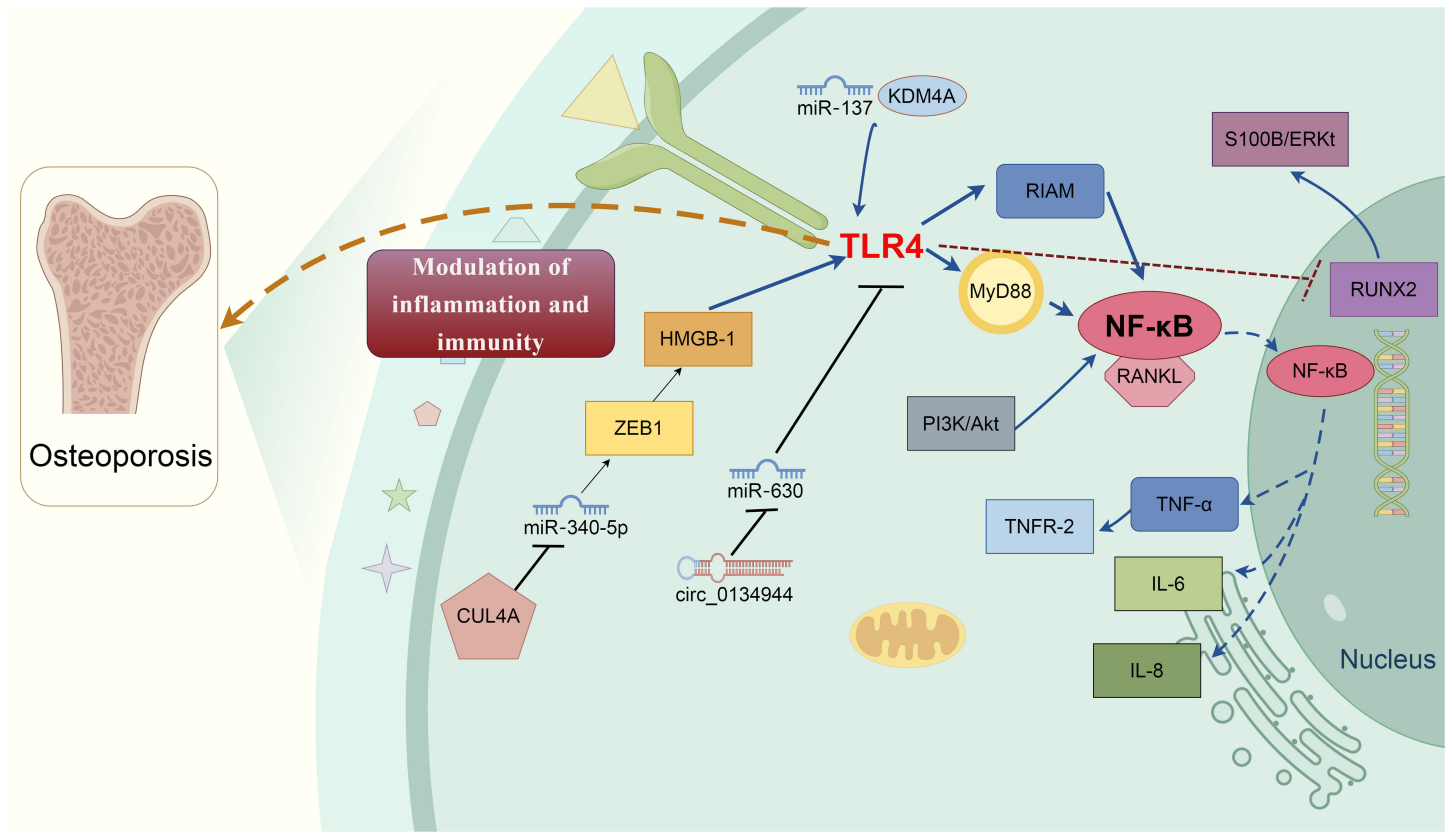
Molecular mechanisms of TLR4 in osteoporosis development. Multifactorial proteins and signaling cascades-mediated by TLR4 are involved in osteoporosis development. Various molecules directly or indirectly involve TLR4-mediated bone remodeling process via cross-talking interaction. For example, E3 ubiquitin ligase CUL4A promotes osteoclast differentiation by upregulating ZEB1 to repress miR-340-5p expression, causing HMGB1 upregulation and the TLR4 activation. Circ_0134944/miR-630 and miR-137/KDM4A elevate the TLR4 expression, together contribute to the pathophysiologic mechanism of osteoporosis. TLR4 induces osteoblast dysfunction by inhibiting the expression of RUNX2 and activating the S100B/ERK signaling pathway. TLR4/MyD88/NF-κB signaling induces the production of multiple proinflammatory cytokines, i.e., TNF-α, IL-1, and IL-6. TLR4, Toll-like receptor 4; NF-κB, nuclear factor kappa B MyD88, Myeloid differentiation primary response gene 88; ERK, Extracellular regulated protein kinases; RANKL, Nuclear factor-kappaB ligand; CUL4A, Cullin 4 A HMGB1, High mobility group box 1; Runx2, Runt-related transcription factor 2; KDM4A, Lysine-specific demethylase 4a.

Six common life-science databases (PubMed, EMBASE, Scopus, Web of Science, Google Scholar, and Cochrane Library) were applied to identify those studies that met the review criteria. In the MEDLINE database, we searched the eligible studies by using the following keywords: ((((((((“Toll-Like Receptor 4”[Mesh]) OR (TLR4)) OR (TLR-4)) OR (Toll Like Receptor 4)) OR (Toll-4 Receptor)) OR (Toll 4 Receptor)) OR (TLR4 Receptor)) OR (Receptor, TLR4)) AND ((((“Osteoporosis”[Mesh]) OR (Osteoporoses)) OR (Bone Loss)) OR (Bone Losses)). Reference lists from related publications were also retrieved in order to identify more relevant studies. As shown in **Figure 1** with the flow diagram, thirty studies were finally included. Among them, six included studies (39, 41–45) provided the clinical data from osteoporosis patients (**Table 1**), 17 studies (12, 37, 38, 40, 46–58) reported the molecular mechanisms underlying the role of TLR4 in osteoporosis (**Table 2**), and seven studies (59–65) provided the data of specific treatments-targeted TLR4 for osteoporosis (**Table 3**). The key information of the 30 eligible studies included the study object, disease modeling method, the status of TLR4, effect on osteoblast or osteoclast, associated genes or pathways, and the main findings of the independent study.

**Table 1:** Studies reported the clinical findings the clinical significance of TLR4 in osteoporosis.

Study	Clinical sample size	Intervention or treatment	Status of TLR4	Associated genes or pathways	Main findings
Santos et al. (41),	227 postmenopausal women (61–95 years)	No	TLR4 polymorphisms	NA	Although TLR4 being candidate gene for osteoporosis in the elderly women, the frequency of TLR4 Asp299Gly and TLR4 Thr399Ile allelic variants were low in postmenopausal women.
Uzar et al. (42),	424 postmenopausal women (58 ± 6 years)	No	TLR4 polymorphisms	NA	8993C>T TLR4 polymorphisms involved in the etiology of postmenopausal osteoporosis.
Kaleta et al. (43),	103 postmenopausal women (51-85 years)	No	TLR4 polymorphisms	NA	TLR4 C1196T polymorphism was not associated with bone mineral density and the incidence of fracture in osteoporotic women after menopause.
Hong et al. (44),	707 participants and human osteoblasts	No	Inhibited	Regulated by miR-34a	rs1057317 polymorphism in TLR4 3’-UTR inhibited the expression of TLR4 by miR-34a, which increased the susceptibility to osteoporosis.
Mi et al. (45),	80 fracture patients and bone marrow stromal cells	SARS-CoV-2-	Inhibited	Upregulated miR-4485	Overexpression of miR-4485-3p suppressed osteogenic differentiation (impairment fracture healing) by inhibiting TLR4 expression.
Yang et al. (39),	306 postmenopausal female subjects	No	Activated	Increased circ_0134944 and inhibited miR-630	Osteoporosis was positively associated with the expression of circ_0134944 and TLR4 and negatively correlated to the expression of miR-630 in the blood and bone tissues samples.

TLR4, Toll-like receptor 4; NA, Not available.

**Table 2:** Studies reported the molecular mechanisms underlying TLR4-mediated osteoporosis.

Study	Study object	Disease modeling method	Status of TLR4	Effect on osteoblast or osteoclast	Associated genes or pathways	Main findings
Itoh et al. (46),	Osteoclasts and bone marrow macrophages	LPS	Activated	Promoted osteoclast	Down-regulated I-kB and up-regulated PI-3 kinase/Akt and NF-κB	LPS enhanced the survival of osteoclasts by promoting TLR4, but not cytokine production.
Johnson et al. (47),	C57Bl/10ScNCr mice	No	Mutated	NA	TLR4/CD14 complexes	Mice harboring mutations in TLR4/CD14 increased bone mineral content.
Kim et al. (48),	Osteoblast-lineage cells	LPS	Activated	Inhibited osteoblastic cells	Up-regulation of TLR-2 and HSP60	Menopause could elevate the expression of HSP-60, resulting in the apoptosis of osteoblast-lineage cells through up-regulation of toll-like receptors.
Xing et al. (50),	Pre-osteoblast cell line	LPS	Activated	Promoted osteoclast	Up-regulated CXCR4 via TLR4	CXCR4/TLR4 involved in LPS-induced bone resorption.
Gillet et al. (51),	MSC-derived osteoblastic cells	Palmitate	Activated	Inhibited osteoblastic cells	Activation of the NF-κB and ERK pathways	Palmitate impaired bone formation by increasing the expression and secretion of IL-6 and IL-8 by the up-regulated of TLR-4 expression. Oleate exerted a protective action on bone cells by inhibiting proinflammatory response and TLR-4 expression.
Liu et al. (52),	Osteoblastic cell line	LPS	Activated	Inhibited osteoblastic cells	Inhibited the expression of ALP, OCN, and Runx2.	LPS inhibited osteoblast differentiation in a TLR4-dependent manner.
Wang et al. (49),	RAW264.7 cells	No	Activated	Promoted osteoclast	Inhibited miR-218 and miR-618	miR-218 and miR-618 involved in the osteoclastogenesis of RAW264.7 cells by modulating the TLR-4/MyD88/NF-κB signaling pathway.
AlQranei et al. (53),	RAW 264.7 cells	LPS	Activated	Promoted osteoclast	Activated the LPS/TLR4/TNF-α/TNFR-2 axis	LPS enhanced osteoclastogenesis in RANKL-primed cells by regulating TLR4/TNF-α axis rather than RANKL/RANK/OPG axis.
Cao et al. (54),	TLR4 knockout C57BL/6 mice	Streptozotocin (STZ)	Activated	Promoted osteoclast	Activated the receptor activator of NF-kB ligand (RANKL).	TLR4 deletion improved STZ-induced diabetic osteoporosis by inhibiting the expression of RANKL.
Liang et al. (37),	MC3T3-E1 cells and TLR4-knockout diabetic rat	Glycolipid	Activated	Inhibited osteoblastic cells	Inhibited the expression of Runx2 and activated S100B/ERK pathway	TLR4 induced the osteoblast dysfunction by inhibiting the expression of Runx2 and activating S100B/ERK signaling pathway.
Oh et al. (55),	Bioinformatics	No	NA	NA	MD2-TLR4-IN-1	MD2-TLR4-IN-1 is an active ligand against comorbidity of osteoporosis.
Xm et al. (56),	SD rats	Streptozotocin (STZ)	Activated	Inhibited osteoblastic cells	Promoted nuclear RIAM–NF-κB expression	TLR4 inhibition improved glucolipotoxicity-induced differentiation in osteoblasts via RIAM regulation of NF-κB nuclear translocation.
Yu et al. (58),	HMGB-1 down female rats with ovariectomy	No	Activated	Inhibited osteoblastic cells	Upregulated HMGB-1 and NF-κB signaling pathway	HMGB-1 knockout suppressed ovariectomy-evoked activation of the TLR4/NF-κB signaling cascade, resulting in the alleviation of osteoporosis.
Chen et al. (38),	C57BL6/J mice	No	Activated	Promoted osteoclast	Upregulated ZEB1 and HMGB1 and inhibited miR-340-5p	E3 ubiquitin ligase CUL4A promoted osteoclast differentiation and osteoporosis by upregulating ZEB1 to repress miR-340-5p expression, causing HMGB1 upregulation and the TLR4 activation.
Rajpar et al. (40),	TLR-4 conditional knockout mice	No	NA	NA	Activated TLR4-NGF-TrkA signaling axis	TLR4 signaling in mature osteoblasts to support load-induced bone formation.
Shen et al. (57),	SD rats	STZ	Activated	Promoted osteoclast	Upregulated the methylation levels of JAK1	TLR4 knockout prevented bone loss in diabetic rats by inhibiting osteoclast hyperactivity, which might be mediated by the regulation of obesity-associated protein-mediated m6^A^ modification.
Yu et al. (12),	Human BMSCs	No	Activated	Promoted osteoclast	Positively correlated with miR-137 expression but negatively correlated with the expression of KDM4A.	MiR−137/KDM4A axis suppressed the osteogenic differentiation of human BMSCs and exacerbating osteoporosis by activating TLR4/NF−κB pathway.

**Table 3:** Studies reported the treatments targeted by TLR4 in osteoporosis.

Study	Study object	Intervention or treatment	Status of TLR4	Effect on osteoblast or osteoclast	Associated genes or pathways	Main findings
Vijayan et al. (59),	Female Sprague-Dawley rats	Methionine	Inhibited	Inhibited osteoclast	TLR-4/MyD88/NF-κB pathway	Methionine inhibited the growth of osteoclast by suppressing TLR-4/MyD88/NF-κB pathway.
Tao et al. (60),	Osteoclasts and bone marrow-derived macrophages; ovariectomy-induced osteoporosis (OVX) mouse model	Dioscin	Inhibited	Promoted osteoblast	Inhibited TLR4/MyD88 pathway to decreased TRAF6 expression	Dioscin against OVX-induced bone loss by promoting osteoblastogenesis and suppressing osteoclastogenesis.
Zeng et al. (62),	RAW264.7 cells and mice	Artesunate	Inhibited	Inhibited osteoclastogenesis	Suppressed TLR4/TRAF6 and the PLCγ1-Ca2+-NFATc1 signaling cascade	Artesunate reduced the bone loss caused by gram-negative bacteria infection in a RANKL-independent manner.
Zhang et al. (63),	Ovariectomized rats	AEG-1 deletion	Inhibited	NA	Inhibited the TLR4/MyD88/NF-κB signaling cascade.	AEG-1 deletion improved bone remodeling in an osteoporosis animal model by inhibiting TLR4/MyD88/NF-κB pathway.
Lu et al. (64),	Bone marrow-derived macrophages	Patchouli Alcohol	Inhibited	Inhibited osteoclast	Downregulated TLR4/Myd88/TRAF6 via PXR and inhibited NF-κB signaling	Patchouli alcohol suppressed osteoclastogenesis by inhibiting RANKL-induced NF-κB signaling cascade.
Xie et al. (61),	MC3T3-E1 cells and bone marrow-derived macrophages	miR-1906 mimic	Inhibited	Inhibited osteoclast	Down-regulating the TLR4/MyD88/NF-kB cascade	miR-1906 mimic reduced bone loss of osteoporosis animal model by down-regulating the TLR4/MyD88/NF-kB pathway.
Yuan et al. (65),	Female ovariectomized C57/BL6 mice	Bacteroides vulgatus	Inhibited	NA	Inhibited TLR-4/p-NF-κB pathway	Bacteroides vulgatus reduced the colonic microbiota dysbiosis by down-regulating the colonic LPS/TLR-4/p-NF-κB pathway.

TLR4, Toll-like receptor 4; BMSCs, Bone marrow mesenchymal stem cells; PXR, Pregnane X receptor; LPS, Lipopolysaccharide; ERK, Extracellular signal regulated kinase; MAPK, Mitogen-activated protein kinase; PI-3, Phosphatidylinositol-3; RANK, Receptor activator of NF-κB; RANKL,RANK ligand; TRAF, TNFR-associated factor; TRAP, Tartrate-resistant acid phosphatase; NF-κB, Nuclear factor kappa B; MyD88, Myeloid differentiation primary response gene 88.

## Clinical significances of TLR4 in osteoporosis

There are six included studies (6/30, 20%) that provided the clinical outcomes of the expression of TLR4 in osteoporosis (**Table 1**). Four of them reported that the TLR4 polymorphisms were associated with the susceptibility to osteoporosis. Many single nucleotide polymorphisms (SNPs) of the TLR4 gene have been detected in various diseases. And some of the SNPs have been reported to be correlated with hyporesponsiveness to inhaled LPS. As aforementioned, TLR4 exerts the defense against microorganisms by recognizing the LPS of the gram-negative bacteria (66). Santos et al. (41) investigated the allele/genotype frequencies and linkage disequilibrium measures of polymorphisms of the TLR4 gene in 227 postmenopausal women aged 61–95 years. They found that although TLR4 was a candidate gene for osteoporosis in elderly women, the frequency of TLR4 Asp299Gly and TLR4 Thr399Ile allelic variants was low in postmenopausal women. This study implied that TLR4 polymorphisms (Asp299Gly and Thr399Ile) did not show a major influence on bone mineral density and osteoporosis status in elderly women. In line with this finding, Kaleta et al. (43) also found that TLR4 C1196T polymorphism was not associated with bone mineral density and the incidence of fracture in osteoporotic women after menopause. They observed that the C1196T genotype frequencies were comparable in the osteoporotic group and the control group (CC: 88% vs 86%; CT: 12% vs 14%, all P>0.05).

However, a large sample clinical study with 424 postmenopausal women (58 ± 6 years) in Poland demonstrated that 8993C>T TLR4 polymorphisms were involved in the etiology of postmenopausal osteoporosis (42). This study showed that 8993C>T TLR4 polymorphisms in osteoporosis and control group were with statistically significant differences, including overrepresentation of heterozygous 8993CT genotype and mutated 8993T allele, and higher frequency of heterozygous 8993CT genotype and mutated 8993T allele. Also, in the Chinese samples, Hong et al. (44) reported that patients with rs1057317 polymorphism in TLR4 increased their susceptibility to osteoporosis, which might be correlated to an inhibitory effect on the expression of TLR4 3’-UTR by miR-34a.

In the molecular mechanism aspect, Yang et al. (39) investigated the association between the expression of circRNAs, miRNAs, and the target genes and TLR4 expression in the blood and bone tissue sample of 306 postmenopausal female subjects. The results revealed that osteoporosis was positively associated with the expression of circ_0134944 and TLR4 and negatively correlated to the expression of miR-630 in the blood and bone tissue samples. This study demonstrated that dysregulation of circRNA-0134944 and TLR4 contributed to the severity of osteoporosis and the risk of osteoporotic fracture by modulating their specific signaling. Interestingly, Levy et al. (67) also showed that TLR4 and its response pathways were associated with the process of trauma-induced bone fracture. The severe acute respiratory syndrome coronavirus 2 (SARS-CoV-2) is currently controlled worldwide. Now, research has begun to evaluate the secondary organ damage caused by COVID-19. Biological alteration of bone remodeling was found in patients who were previously infected with COVID-19 (68). Mi et al. (45) reported that overexpression of miR-4485-3p suppressed osteogenic differentiation (impairment fracture healing) in COVID-19 patients, while TLR-4 was the key potential target of miR-4485. This study highlighted miR-4485-3p/TLR4 axis might provide a therapeutic direction for anti-osteoporosis therapy in patients with a prior infection of COVID-19.

Postmenopausal osteoporosis is a systemic bone disease characterized by low bone mass after menopause. It was reported that activation of estrogen receptor α (ERα) in the postmenopausal stage could inhibit TLR4 signaling and macrophage-related inflammation (69). Consistently, Guo et al. showed that 17 β-estradiol (E2) suppressed the release of inflammatory factors via inhibiting TLR4 (70). Therefore, there is a negative relationship between estrogen and TLR4 expression. Since postmenopausal osteoporosis may be partially induced by low estrogen, estrogen-deficient may up-regulate the level of TLR4, thereby indicating the pivotal role of TLR4 in osteoporosis. In the included studies of this review, Kaleta et al. (43) reported that TLR4 C1196T polymorphism is related to bone mineral density and fracture incidence in osteoporotic women after menopause. In line with this study, Uzar et al. (42) also demonstrated that mutated 8993T allele of 8993C>T TLR4 polymorphisms played a pivotal role in the etiology of postmenopausal osteoporosis. Consistently, TLR4 Asp299Gly or TLR4 Thr399Ile polymorphisms have a major influence on adiposity, bone mineral density or osteoporosis status in elderly women (41). An experimental study conducted by Kim et al. (48) showed that HSP60/TLR4 played a critical role in promoting bone loss in the estrogen-deficient state (namely postmenopausal osteoporosis). These studies shed light on a possible therapeutic strategy targeting TLR4 for the treatment of postmenopausal estrogen-deficient osteoporosis.

Taken together, the above clinical studies demonstrate that the clinical significance of TLR4 polymorphism in osteoporosis is still being debated. Mechanistically, non-coding RNAs (i.e., circ_0134944 and miR-4485-3p) play an essential role in TLR4-mediated osteoporosis.

## Molecular mechanisms of TLR4 in osteoporosis development

Since mounting clinical and experimental studies have investigated the potential role of TLR4 in osteoporosis, a better understanding of the biological function of TLR4 and its associated pathways on osteoporosis development is profound for the researchers. **Table 2** illustrates the potential molecular mechanisms underlying the biological functions of TLR4 in osteoporosis, which was derived from 17 included studies.

## Role of the TLR4/NF-κB pathway in osteoporosis

TLR4/NF-kB signaling pathway is an important pathway mediating apoptosis and inflammatory activation (71), which can affect bone formation, osteoclastic resorption, and inflammatory response. TLR4 is highly expressed in human bone marrow. Human and mouse MSCs are the precursor cells of osteoblasts and adipocytes, expressing TLR4 mRNA (72). TLR4 signaling mainly depends on myeloid differentiation primary response gene 88 (MyD88) and mediates NF-κB activation downstream of the TLR4 signaling pathway, which promotes osteoclastogenesis and differentiation and participates in osteoblast migration (73). NF-κB is a central regulator of the inflammatory response and determines whether inflammation subsides or develops into a cascade of injury (74). NF-κB inhibition attenuates the severity of the injury and immune response by down-regulating the overexpression of cytokines (IL-6 and TNF-α). It has been shown that TLR4 deficiency attenuates the inflammatory response in the NF-κB pathway, thereby improving osteoporosis (54).

Seven included studies reported the effects of TLR4/NF-κB signaling in osteoporosis development. Itoh et al. (46) showed that LPS enhanced the survival of osteoclasts by promoting TLR4/NF-κB, activating PI-3 kinase/Akt pathways, and down-regulating I-kB, but not cytokine production. Streptozotocin (STZ) is a common method for establishing a diabetes model. Cao et al. (54) demonstrated that STZ induced osteoporosis by activating TLR4 expression and the receptor activator of NF-kB ligand (RANKL) through the promotion of osteoclast. They further found that TLR4 deletion improved STZ-induced diabetic osteoporosis by inhibiting the expression of RANKL. In line with this finding, Shen et al. (57) revealed that STZ caused osteoporosis by inhibiting osteoblastic cells, which might be mediated by promoting TLR4 and nuclear RIAM–NF-κB expression. In the subsequent experiment, the authors observed that TLR4 inhibition improved glucolipotoxicity-induced differentiation in osteoblasts via RIAM regulation of NF-κB nuclear translocation. Yu et al. (58) applied ovariectomy to establish the female osteoporosis rat model. The results of their study showed that high mobility group box 1 (HMGB-1) knockout suppressed ovariectomy-evoked activation of the TLR4/NF-κB signaling cascade, resulting in the alleviation of osteoporosis. Gillet et al. (51) reported that palmitate-impaired bone formation (characterized by the inhibition of osteoblastic cells) increased the expression and secretion of IL-6 and IL-8 by the up-regulation of TLR-4 expression. In addition, the authors also found that oleate exerted a protective action on bone cells by inhibiting proinflammatory response and TLR-4 expression. A more recent study (12) showed that the miR−137/KDM4A axis suppressed the osteogenic differentiation of human BMSCs (promoted osteoclast) and exacerbated osteoporosis by activating the TLR4/NF−κB pathway.

MyD88 and TIR-domain-containing adapter-inducing interferon-β (TRIF) serve as the primary adaptor proteins in Toll-like receptor signaling, facilitating the downstream pathway (75). Additionally, Mal (MyD88-adapter-like) is crucial for TLR-4 signal transduction. The interaction between TLR4 and MyD88 is responsible for the activation of downstream signals, thus contributing to bone remodeling. A previous study has demonstrated the essential role of the TLR-4/MyD88/NF-κB signaling pathway in osteoclast development (59). It was reported that inhibiting TLR4/MyD88 signaling in osteoclast precursors effectively mitigates the progression of osteoporosis and subsequently bone loss (59). Zhang et al. (63) conducted a study on ovariectomized rats with osteoporosis and found that TLR4/MyD88 has the ability to regulate bone remodeling-related cytokines. The researchers observed that ovariectomy led to the activation of the TLR4/MyD88/NF-κB pathway, but this effect was nullified when HMGB-1 was knocked down (58), miR-1906 was mimicked (61), or AEG-1 was deleted (63). Inhibition of the TLR4/MyD88/NF-κB pathway demonstrated positive outcomes such as mitigating bone histopathological damage, reducing bone loss, and promoting osteogenesis. Consequently, targeting the TLR4/MyD88/NF-κB pathway may offer promising prospects for the development of new therapeutic interventions.

The above studies showed that the TLR4/NF-κB pathway is closely related to the development of osteoporosis. TLR4, as a key receptor in the inflammatory signaling pathway, is involved in influencing bone formation and bone resorption. The activation of the TLR/NF-κB signaling pathway leads to osteoclast differentiation and maturation, which in turn accelerates bone destruction, while inhibition of the TLR4/NF-κB pathway could inhibit the differentiation and maturation of osteoclasts and regulate osteoblast differentiation, thus attenuating the occurrence of osteoporosis.

## Roles of TRIF-TRAM-IRF3 pathway and AP-1 in osteoporosis

MyD88 and TIR-domain-containing adapter-inducing interferon-β (TRIF) are the two main adaptor proteins of Toll-like receptor signaling that mediate downstream pathways. TLR4 exerting its biological function mainly depends on two signal-transduction cascades, TIRAP and MyD88 for regulating proinflammatory cytokines and TRAM and TRIF for driving the production of type I interferons (76). TLR4 interacted with MyD88 for its downstream signal activation, together contributing to bone remodeling. A previous study indicated that TLR-4/MyD88/NF-κB signaling pathway was integral for osteoclast development (59). TLR4 can activate transcription factors NF−κB and AP−1, thus promoting the production of inflammatory cytokines (77). TLR4/MyD88/AP-1 pathway has been found to regulate the expression of inflammatory cytokines, thereby involving the development of multiple diseases (78, 79). Also, this cascade plays a key role in osteoclasts activation. AP-1 has been found to be associated with musculoskeletal health (i.e., skeletal muscle and myogenic differentiation) (80, 81). Tao et al. (60) reported that dioscin could reduce ovariectomy-induced bone loss by regulating both TLR4/MyD88 and MAPKs/AP-1 pathways as well as blocking NF-κB and AP-1 nuclear translocations. This is the only study that reported the role of TLR4 and AP-1 in osteoporosis. Therefore, the exact role of TRIF-TRAM-IRF3 and the MAPKs/AP-1 pathways in the TLR4-related osteoporosis needs further investigation.

## Roles of inflammatory factors in osteoporosis

Inflammation is closely related to bone metabolism. Chronic inflammation and inflammatory environment are the common causes of bone loss (82). Innate immune responses are involved in the development and maintenance of inflammation. Inflammation responses can be regulated by a family of toll-like receptors, among which TLR4 receptors are particularly prominent due to their expression in the musculoskeletal system (29). It is reported that inflammation upregulates TLR4 expression in various cells and tissues and enhances cellular immunity, while inhibition or knockdown of the TLR4 receptor effectively reduces inflammatory responses and cellular immunity (83).

TNF-α and IL-6 play important roles in immune response and bone metabolism (84). Both of the two proinflammatory cytokines enhance macrophage activation and antigen presentation and modulate immunity through different mechanisms (85). Besides, they are important pathogenic factors associated with immune-mediated bone diseases such as rheumatoid arthritis and osteoporosis. AlQranei et al. (53) reported that LPS enhanced osteoclastogenesis in RANKL-primed cells by regulating the TLR4/TNF-α axis rather than the RANKL/RANK/OPG axis. Activated the LPS/TLR4/TNF-α/TNFR-2 axis was found to promote the growth of osteoclast (53). Studies have reported that TNF-α inhibits osteoblast activity at certain stages of differentiation and stimulates osteoclast proliferation and differentiation. Gillet et al. (51) showed that high levels of IL-6 and IL-8 could be induced by high expression of TLR4, resulting in the inhibition of osteoblastic cells. Intriguingly, the authors further observed that oleate could reverse this phenomenon by suppressing the pro-inflammatory response and TLR-4 expression. IL-6 is a major inflammatory response factor associated with bone disease. High levels of IL-6 can bind to osteoclast receptors, promote bone catabolism, and increase the rate of bone resorption rate, which plays a key role in joint inflammation and destruction (86). The above studies reveal that relieving inflammation can improve osteoporosis and bone metabolism disorders, which can be implemented by TNF-α inhibitor, IL-6 inhibitor, or TLR-4 inhibitor treatment.

## Roles of TLR4 on immune cells influences osteoporosis

Bone loss can be induced by the infection of pathogenic bacteria and their recognition by the host immune system (87). TLR4 is highly expressed by immune cells, which can trigger immune responses after the detection of endogenous ligands (88). TLR4 is a pivotal pattern recognition receptor correlated to innate immunity, functioning to mediate glucolipid toxicity (22). TLR4-mediated NF-κB signaling is found to trigger inflammatory response (89). TLR4 regulates the bone mineral content and bone metabolism of mammals. The host innate immune response to pathogens is mediated primarily by TLR signaling (i.e., TLR2 and TLR4) (90). It has been shown that LPS affects bone resorption by TLR4-mediated immune response. Bacterial LPS cause innate immune response by TLR4/LY96 and CD14 (91). TLR4 is a key regulator of immune responses during chronic inflammation and postmenopausal osteoporosis in the postmenopausal females (39). Specific treatment balances inflammatory pathways to maintain immunity by blockading of the TLR4 signaling pathway.

## Roles of non-coding RNAs in osteoporosis

Non-coding RNAs (ncRNAs), a classification of RNA molecules, function to regulate various biological processes, i.e., gene expression, cell growth, differentiation, and cell death (92). Among them, microRNAs are the most studied RNA that exert a variety of biological functions in multiple diseases (93). There are three included studies that reported the roles of ncRNAs-mediated TLR4 in osteoporosis. Chen et al. (38) reported that E3 ubiquitin ligase CUL4A promoted osteoclast differentiation by upregulating ZEB1 to repress miR-340-5p expression, causing HMGB1 upregulation and the TLR4 activation, thus inducing the development of osteoporosis. Wang et al. (49) showed that both miR-218 and miR-618 were involved in the osteoclastogenesis of RAW264.7 cells, which might be associated with the modulation of the TLR-4/MyD88/NF-κB signaling cascade. This study further proved that TLR4 was the direct target of miR-218 and miR-618 via the bioinformatics analysis and luciferase reporter assay. In line with the above included studies, a more recent study conducted by Yu et al. (12) demonstrated that the miR−137/KDM4A axis suppressed the osteogenic differentiation of human BMSCs and exacerbated osteoporosis by activating TLR4/NF−κB pathway. In a clinical study developed by Yang et al. (39), it was reported that circ_0134944, miR-630, and TLR4 together contributed to the pathophysiologic mechanism of osteoporosis. In summary, ncRNAs exhibit a key role in osteoporosis by regulating the expression of TLR4.

## Role of RUNX2 in osteoporosis

Runt-related transcription factor 2 (RUNX2) is a master transcription factor of osteoblast differentiation (94). It has been suggested to be a pivotal factor in the development of osteoporosis by aggravating bone mineral density. Liu et al. (52) reported that LPS could inhibit the differentiation of osteoblastic cells by inhibiting the expression of ALP, OCN, and RUNX2 in a TLR4-dependent manner (activated TLR4). Glycolipid is found to suppress the growth of osteoblasts, thus it can be applied to establish the disease model of osteoporosis or bone loss. Liang et al. (37) implied that TLR4 might induce osteoblast dysfunction by inhibiting the expression of RUNX2 and activating the S100B/ERK signaling pathway. The above two studies demonstrated that TLR4 expression was negatively correlated with the expression of RUNX2, functioning together with osteoporosis development.

## Role of CD14 in osteoporosis

CD14, a glycosylphosphatidylinositol-anchored molecule, is a cell surface co-receptor for TLR4. Previous study demonstrates that CD14 controls the signaling functions of TLR4 (95). Both TLR4 and CD14 are involved in LPS-signaling process as well as play an important role in bone mineral content (96, 97). LPS-induced inflammatory responses may be greatly declined when lacking of either CD14 or TLR4. Itoh et al. (46) reported that TLR4 and CD14 mRNAs were expressed in osteoclasts as well as the precursors of osteoclasts (bone marrow macrophages). Johnson et al. (47) demonstrated that TLR4 and CD14 were involved in the metabolism of both bone mineralization and adiposity. The authors found that CD14 knockout mice had elevated bone area, bone mineral content, and bone density (all P<0.001). They further showed that TLR4/CD14 complexes not only functioned in inflammation and immunity but also correlated with the regulation of bone mineralization. Since CD14 can initiate the signal transduction through TLR4 during the inflammatory response, abnormal TLR4 expression-associated osteoporosis may be partially mediated by the dysregulation of CD14.

## Summary of the role of TLR4 in osteoblasts and osteoclasts

TLR4, a receptor found on the surface of osteoclasts and osteoblasts, plays a crucial role in regulating the immune response in individuals with chronic inflammation, ultimately leading to the development of osteoporosis (58). Both human and mouse MSCs, serving as progenitor cells for osteoblasts and adipocytes, express TLR4 mRNA. Furthermore, TLR4 activation in MSCs, osteoblasts, and osteoclasts is implicated in both catabolic and anabolic processes (29). The TLR4/NF-kB signaling pathway serves as a significant mediator of apoptosis and inflammatory activation, exerting an impact on bone formation, osteoclastic resorption, and the inflammatory response (98). The TLR4 signaling cascade is primarily reliant on MyD88 and facilitates NF-κB activation, thereby facilitating osteoclastogenesis and differentiation and contributing to osteoblast migration. The activation of the TLR4/NF-κB signaling pathway ultimately leads to the differentiation and maturation of osteoclasts, consequently hastening bone degradation. The inhibition of the TLR4/NF-κB pathway has the potential to impede the differentiation and maturation of osteoclasts, as well as regulate osteoblast differentiation, thereby mitigating the development of osteoporosis (54). The absence of TLR4 expression has been shown to prevent bone loss by suppressing osteoclast hyperactivity (57). Consequently, based on the aforementioned evidence, the administration of a TLR4 inhibitor to hinder progressive osteoclast hyperactivity could serve as a promising therapeutic approach for the treatment of osteoporosis.

## Other potential mechanisms

In addition to the TLR4/NF-κB pathway, proinflammatory effects, ncRNAs, and RUNX2, some other potential molecular mechanisms are also proposed for TLR4-mediated osteoporosis. Johnson et al. reported that C57Bl/10ScNCr mice harboring mutations in TLR4/CD14 complexes might increase bone mineral content. Kim et al. (48) LPS inhibited the differentiation of osteoblastic cells by activating the TLR4, TLR-2, and HSP60. The authors indicated that menopause could elevate the expression of HSP-60, causing the apoptosis of osteoblast-lineage cells through up-regulation of toll-like receptors. Xing et al. (50) showed that the activation of CXCR4/TLR4 contributed to LPS-induced bone resorption. Oh et al. (55) found that the MD2-TLR4-IN-1 pathway was an active ligand against the comorbidity of osteoporosis by conducting a bioinformatic analysis. In a SD rat model of STZ, Xm et al. (56) demonstrated that TLR4 knockout prevented bone loss in diabetic rats by inhibiting osteoclast hyperactivity, which might be mediated by the regulation of obesity-associated protein-mediated m6A modification (inhibited the methylation levels of JAK1). Taken together, the above several different mechanisms are reported to be involved in the development of TLR4-related osteoporosis. However, the exact underlying mechanism is still unclear, which is needed to further explore by future studies.

**Figure 1** showed the underlying molecular mechanisms of TLR4-mediated bone remodeling of osteoporosis.

## Roles of TLR4 in aging-related osteoporosis

The prevalence of osteoporosis among the elderly is rising annually due to the escalating aging trend within the population (99). Both men and women experience bone loss as a result of aging, which is primarily caused by hormonal alterations and age-related dysfunction of osteoblasts (100). Dysregulation of the tissue microenvironment and impaired functioning of specific stem cells are two prominent changes associated with aging. Within the aging microenvironment, BMSCs not only serve as effector cells but also modulate the immune response and alter the microenvironment. Interleukin-6 (IL-6) serves as a key mediator in the initiation of inflammatory responses linked to bone diseases. Activation of the TLR4 and AKT pathway by IL-6, along with the inhibition of Setd7 expression, has been observed (101). Moreover, the suppression of Setd7 expression in the bone tissues of aged mice suggests its potential as a promising molecular target for the prevention and treatment of age-related osteoporosis. Notably, postmenopausal osteoporosis, a systemic bone disease characterized by reduced bone mass following menopause, represents one manifestation of the aging process in women. Kaleta et al. (43) conducted a study that found a correlation between the TLR4 C1196T polymorphism and bone mineral density as well as fracture incidence in postmenopausal women with osteoporosis. Similarly, Uzar et al. (42) demonstrated that the mutated 8993T allele of the 8993C>T TLR4 polymorphisms played a significant role in the development of postmenopausal osteoporosis. Furthermore, TLR4 Asp299Gly or TLR4 Thr399Ile polymorphisms were found to have a substantial impact on adiposity, bone mineral density, and osteoporosis status in elderly women (41). Additionally, an experimental study conducted by Kim et al. (48) revealed that HSP60/TLR4 signaling pathway contributes to the promotion of bone loss in the estrogen-deficient state (namely postmenopausal osteoporosis).

## Current treatments for osteoporosis by targeting TLR4

In this review, seven included studies reported specific treatments for osteoporosis by targeting TLR4. All these studies discovered that inhibition of TLR4 was one of the important mechanisms underlying the anti-osteoporotic effects exerted by specific treatments. Methionine is a sulphur-containing essential amino acid. It functions to synthesize cartilage and is thus applied for the treatment of arthritis and osteoporosis (102, 103). Vijayan et al. (59) reported that methionine inhibited the growth of osteoclast by suppressing the TLR-4/MyD88/NF-κB pathway. Dioscin, a natural steroidal saponin, has been found to exhibit hepatoprotective, anti-ischemia reperfusion injury, and anti-obesity effects (104, 105). Tao et al. (60) showed that dioscin against OVX-induced bone loss by promoting osteoblastogenesis and suppressing osteoclastogenesis by inhibiting the TLR4/MyD88 pathway and decreasing TRAF6 expression. As aforementioned, ncRNAs play a key role in TLR4-mediated osteoporosis. Xie et al. (61) investigated the effect of microRNA-related intervention in the expression of RLR4 and biological alternation of osteoclast in MC3T3-E1 cells and bone marrow-derived macrophages. The authors found that miR-1906 mimic significantly reduced bone loss in osteoporosis animal models by down-regulating the TLR4/MyD88/NF-kB pathway (61). Artesunate, a semisynthetic derivative of artemisinin, has been commonly applied to treat falciparum malaria with little toxicity (106). Zeng et al. (62) demonstrated that artesunate could reduce the bone loss caused by gram-negative bacteria infection by suppressing TLR4/TRAF6 and the PLCγ1-Ca2+-NFATc1 signaling cascade in a RANKL-independent manner. Astrocyte-elevated gene-1 (AEG-1) is a late response gene induced by human immunodeficiency virus, which has multiple pro-tumorigenic functions (i.e., angiogenesis and malignant transformation) (107). In addition to the biological effect on cancers, AEG-1 deletion was found to improve bone remodeling in an osteoporosis animal model by inhibiting the TLR4/MyD88/NF-κB pathway. Patchouli alcohol (PA), a tricyclic sesquiterpene extracted from pogostemon cablin, functions to reduce inflammation in gastrointestinal cells (108). Lu et al. (64) demonstrated that PA suppressed osteoclastogenesis by downregulating TLR4/Myd88/TRAF6 via pregnane X receptor (PXR) and inhibited NF-κB signaling. *Bacteroides vulgatus* is identified to be a probiotic microbiota. It is reported that *Bacteroides vulgatus* combined with *B. dorei, B. vulgatus* can reduce the concentration of serum and fecal LPS, thus preventing cytokine induction (109). In the female ovariectomized C57/BL6 mice models, Yuan et al. (65) found that *Bacteroides vulgatus* reduced the colonic microbiota dysbiosis by down-regulating the colonic LPS/TLR-4/p-NF-κB pathway, resulting in amelioration of lumbar bone loss in ovariectomized mice. Collectively, the seven included studies suggested that interventions targeted with the inhibition of TLR4 exerted a promising anti-osteoporosis effect.

LPS is an important component of the outer membrane of gram-negative bacteria and can induce inflammation (110). As reported, LPS directly causes osteoclast formation and bone resorption independent of the expression of RANKL. TLR4, one of the receptors of LPS, increases in both osteoclast differentiation and survival. Within the topic of this review, three included studies (60, 62, 64) reported the roles of TLR4/TRAF6 in osteoporosis development. He et al. (111) demonstrated that sinomenine decreased the expression of TLR4 and TRAF6 during osteoclast differentiation, leading to a reduction in osteoclastogenesis and osteolysis induced by LPS. Furthermore, sinomenine down-regulated the downstream expression of NF-κB, AP-1, and Ca^2+^/NFATc1 in LPS-induced osteoclastogenesis. Based on the key role of TLR4 expression in inflammation, the authors concluded that gram-negative bacteria and other inflammation-induced osteolysis might suppress the TLR4 and TRAF6 signaling. In line with He et al.’s findings, Ye et al. (112) observed that treatment with berberine hydrochloride led to a reduction in the expression levels of TLR4 and TRAF6 during LPS-induced osteoclastogenesis. The mechanism underlying this effect involves the targeting of TRAF6 and NFATc1 by berberine hydrochloride, resulting in the inhibition of osteoclastogenesis and bone destruction through the suppression of the TRAF6−Ca^2+^−calcineurin−NFATc1 signaling pathway. These findings suggest that the down-regulation of TLR4/TRAF6 expression may significantly mitigate LPS-induced osteoclastogenesis and osteolysis, potentially by interfering with the Ca^2+^/NFATc1 signaling cascades.

Based on the above evidence, several agents have been identified to improve bone metabolism via the inhibition of TLR4, including methionine, dioscin, miR-1906 mimic, artesunate, AEG-1 deletion, patchouli alcohol, and Bacteroides vulgatus. Dioscin, artesunate, and patchouli alcohol are the natural extractives and herbal extracts, which are mainly available for research use. These small molecule compounds have been investigated in numerous *in vitro* and *in vivo* studies but have not direct applications in clinical practice. All the three relevant included studies are experimental designed. Therefore, the therapeutic effects against osteoporosis induced by these natural extractives need to be validated by the phase I clinical trial with clear indications, cycles of use, use dosage, administration methods, and adverse effects. Methionine is an essential amino acid. Symptoms of methionine deficiency include fatigue, muscle weakness, and impaired immune function. The other four agents (i.e., methionine, miR-1906 mimic, AEG-1 deletion, and Bacteroides vulgatus) have been reported to reduce bone loss during osteoporosis. However, all these independent studies are the pre-clinical study and lack of detection of toxigenicity. Therefore, further clinical studies are needed to determine the feasibility of using these agents for treating or preventing osteoporosis, which may maximize the prospects of successful clinical translation.

## Future perspectives

This review highlighted that overactivation of TLR4 has been associated with the development of osteoporosis, while inhibition of TLR4 expression significantly prevented osteoporosis. Previously, Pérez et al. conducted a review focusing on the role of TLR4 on osteoblast metabolism and function. In this review, we are more focused on the intact role of TLR4 in bone remodeling of osteoporosis, with regard to the effects on both osteoblasts and osteoclasts as well as from both experimental and clinical evidence. Due to osteoporosis is not considered to be a self-limited chronic disease, it commonly requires lifelong management (113). Therefore, increasing numbers of research in this field have emerged to explore additional molecular targets for the treatment of osteoporosis. Osteoimmunology has received extensive attention over the past 20 years. Several innate immune cells have emerged as key regulators of osteoporosis by producing proinflammatory mediators and modulating the TLR4/NF-κB as well as the RANK/RANKL axis (114). Since the immune system and the skeletal system share a wide variety of molecules (i.e., cytokines, chemokines, hormones, receptors, and transcription factors) and the close interaction between osteoblasts and immune cells (115), future immunotherapy-targeted TLR4 may provide a promising therapeutic direction against osteoporosis. In addition to the innate immune cells, inflammation-mediated intestinal epithelial cells and T cells, also play important roles in osteoporosis development (116), suggesting anti-inflammatory treatment (i.e., TLR4 inhibitor) can effectively prevent osteoporosis. In-depth studies on the correlation between the TLR4 signaling pathway and osteoporosis may not only help to explain the pathogenesis of osteoporosis but also provide new targets and strategies for the treatment of osteoporosis. TLR4 targeting interventions are now being attempted for vaccination (agonist) and treatment of inflammatory diseases (antagonist) (117). As expected, inhibition of progressive osteoclast hyperactivity by treating with TLR4 inhibitor can be an effective therapeutic strategy for osteoporosis. However, current studies on the specific molecular mechanisms and treatments of TLR4’s involvement in osteoporosis are mostly derived from animal experiments or *in vitro* studies. Clinical research on the potential therapeutic target of TLR4 in osteoporosis is still in its infancy. Based on the **Tables 1**–**3** in this review, both preclinical and clinical studies implied that TLR4 might be a promising therapeutic target. However, TLR4 can be developed into a biotherapeutic drug target and requires further investigation. The long-term effects on human general health by using the TLR4 inhibitor for preventing post-menopausal osteoporosis warrants further study and inquiry. Since TLR4 is a pivotal factor in initiating inflammation and immunity, suppressing TLR4 expression may result in the restraint of inflammation reactions and immune responses. It is important to know that a moderate inflammatory response is a kind of defense response, which is conducive to clearing pathogens and restoring health. On the other hand, long-term immunosuppression could be a predisposing factor for infection, autoimmune disease, inducing malignancy, and other implications. Therefore, further preclinical and clinical studies are warranted to investigate the effectiveness and safety of TLR4 inhibitors for treating post-menopausal osteoporosis.

## Conclusion

This study is the latest comprehensive review focusing solely on the key role of TLR4 in clinical practice and the pathogenesis of osteoporosis. TLR4 polymorphisms and aberrant expression of TLR4 have been associated with the clinical significance of osteoporosis in clinical practice. Mechanistically, dysregulation of osteoblasts and osteoclasts induced by abnormal expression of TLR4 is the main molecular mechanism underlying TLR4-mediated osteoporosis, which may be associated with the interactions between TLR4 and NF-κB pathway, proinflammatory effects, ncRNAs, and RUNX2. *In vivo* and *in vitro* studies demonstrate that many promising substances or agents (i.e., methionine, dioscin, miR-1906 mimic, artesunate, AEG-1 deletion, patchouli alcohol, and *bacteroides vulgatus*) have been able to improve bone metabolism (i.e., inhibits bone resorption and promotes bone formation) via the inhibition of TLR4. According to the above evidence, future therapeutic strategies targeting TLR4 and its associated molecules and signaling cascades might provide a new insight for osteoporosis treatment.

## Author contributions

XZ: Data curation, Methodology, Writing – original draft. LZ: Formal analysis, Project administration, Writing – review & editing. LZD: Data curation, Investigation, Methodology, Writing – review & editing. WX: Formal analysis, Supervision, Writing – original draft. XL: Conceptualization, Investigation, Writing – original draft, Writing – review & editing. LD: Writing – review & editing, Supervision.
